# Why don’t illiterate women in rural, Northern Tanzania, access maternal healthcare?

**DOI:** 10.1186/s12884-021-03906-2

**Published:** 2021-06-28

**Authors:** Dismas Matovelo, Pendo Ndaki, Victoria Yohani, Rose Laisser, Respicious Bakalemwa, Edgar Ndaboine, Zabron Masatu, Magdalena Mwaikambo, Jennifer L. Brenner, Warren M. Wilson

**Affiliations:** 1 Department of Obstetrics & Gynecology, Catholic University of Health and Allied Sciences, Mwanza, Tanzania; 2 Bugando Medical Centre, Mwanza, Tanzania; 3 School of Public Health, Catholic University of Health and Allied Sciences, Mwanza, Tanzania; 4 School of Nursing, Catholic University of Health and Allied Sciences, Mwanza, Tanzania; 5 Department of Pediatrics, Catholic University of Health and Allied Sciences, Mwanza, Tanzania; 6 District Medical Officer, Misungwi District, Mwanza, Tanzania; 7 Agriteam Health Tanzania, Mwanza, Tanzania; 8 Departments of Pediatrics and Community Health Sciences, University of Calgary, Calgary, Canada; 9 Departments of Anthropology & Archaeology and Community Health Sciences, University of Calgary, Calgary, Canada

## Abstract

**Background:**

In 2017, roughly 540 women in Sub-Saharan Africa died every day from preventable causes related to pregnancy and childbirth. To stem this public-health crisis, the WHO recommends a standard continuity of maternal healthcare, yet most women do not receive this care. Surveys suggest that illiteracy limits the uptake of the recommended care, yet little is understood about why this is so. This gap in understanding why healthcare is not sought by illiterate women compromises the ability of public health experts and healthcare providers to provide culturally relevant policy and practice. This study consequently explores the lived experiences related to care-seeking by illiterate women of reproductive age in rural Tanzania to determine why they may not access maternal healthcare services.

**Methods:**

An exploratory, qualitative study was conducted in four communities encompassing eight focus group discussions with 81 illiterate women, 13 in-depth interviews with illiterate women and seven key-informant interviews with members of these communities who have first-hand experience with the decisions made by women concerning maternal care. Interviews were conducted in the informant’s native language. The interviews were coded, then triangulated.

**Results:**

Two themes emerged from the analysis: 1) a communication gap arising from a) the women’s inability to read public-health documents provided by health facilities, and b) healthcare providers speaking a language, Swahili, that these women do not understand, and 2) a dependency by these women on family and neighbors to negotiate these barriers. Notably, these women understood of the potential benefits of maternal healthcare.

**Conclusions:**

These women knew they should receive maternal healthcare but could neither read the public-health messaging provided by the clinics nor understand the language of the healthcare providers. More health needs of this group could be met by developing a protocol for healthcare providers to determine who is illiterate, providing translation services for those unable to speak Swahili, and graphic public health messaging that does not require literacy. A failure to address the needs of this at-risk group will likely mean that they will continue to experience barriers to obtaining maternal care with detrimental health outcomes for both mothers and newborns.

**Supplementary Information:**

The online version contains supplementary material available at 10.1186/s12884-021-03906-2.

## Background

Globally, the maternal mortality ratio declined by 38% between 2000 and 2017. However, in 2017, 810 women died every day from pregnancy- or childbirth-related complications; 94% of these deaths occurred in low and lower middle-income countries (LMICs) [1], most commonly in rural areas [2]. Sub-Saharan Africa (SSA) has the highest regional maternal mortality ratio at 546/100,000, which is three times higher than the next highest region, South Asia (182/100,000). In 2015, Tanzania’s maternal mortality ratio (556/100,000) [3] was the 15^th^ worst among the 46 SSA countries [4]. Moreover, the adult lifetime risk of maternal death of 1 in 36 in SSA contrasts markedly with 1 in 3300 in high-income countries [5].

Common causes of maternal death in SSA include pre-existing disorders such as HIV, anemia, or malaria, exacerbated by pregnancy (28.6%), haemorrhage (24.5%), hypertension (16.0%), sepsis (10.3%), abortion (9.6%), other direct causes (9.0%), and embolism (2.1%) [6]. What is more, the pregnant woman is not the only one at risk; globally, in 2015, 2.6 million babies were stillborn [7] and in 2017, 2.6 million children died in their first month of life, of which roughly 2 million (77%) died within the first six days of life [8], most commonly in least developed countries [9]. In 2018, Tanzania’s neonatal mortality rate was 21.1, in comparison to high-income countries where the rate averages 3.1 [9]. Disparities between least-developed and high-income countries in the maternal mortality ratio, adult lifetime risk of maternal death, and neonatal mortality rates suggest that many more pregnancy-related deaths in least-developed countries could be prevented.

In order to improve maternal and neonatal health, the WHO recommends that pregnant women and neonates receive globally recognized standard of healthcare. This includes a series of antenatal care (ANC) visits [7], giving birth at a health facility with a skilled birth attendant, and postnatal care provided to both a mother her infant [10]. ANC enables early screening and recognition of problems and co-morbidities, prevention of complications, micronutrient supplementation, and, crucially, an opportunity for healthcare providers to communicate with and support women during a critical period [7, 11, 12]. In Tanzania, a woman who attends ANC is twice as likely to deliver in a health facility than a woman with no ANC visits [13]. Giving birth in a health facility with a skilled birth attendant, in turn, reduces stillbirths and deaths from intrapartum–related complications by about 20% [14]. Despite these impactful interventions, only about half of pregnant women globally receive even four ANC visits [11]. Similarly, in Misungwi District, Tanzania only 47% of mothers surveyed in 2016 had attended four or more ANC visits [15] with their last pregnancy and 61% delivered at a health facility [13].

Variables influencing whether pregnant women receive recommended maternal health interventions have been explored in several studies. In both India [16, 17] and SSA [18–20], researchers identified illiteracy (defined as not knowing how to read or write) as an important risk factor for failure to attend ANC and higher maternal mortality. This is consistent with general linkages between low literacy and worse health outcomes [21] and lower utilization of health services by illiterate women [22]. Some studies have identified lower care-seeking due to the stigma associated with illiteracy: some illiterate individuals were hesitant to disclose their illiteracy to healthcare workers, compromising provider support for such individuals [23–25]. Studies elsewhere have documented a positive correlation between level of maternal formal education and perinatal healthcare seeking behaviour [13, 26–33].

Relatedly, a 2016 survey in Tanzania’s Misungwi District, Tanzania [15] documented that 35% of sampled mothers women were illiterate, versus 26.91% for Tanzanian women overall [34]. In this district, 41% of illiterate women report attending ANC at least four times compared with 49% of literate women, a significant difference (2 × 2 χ^2^, *p *= 0.02). And, in comparison to the level of formal education received, number of children, and maternal age, illiteracy was the strongest predictor of having fewer than four ANC visits. Likewise, illiteracy in Misungwi District was significantly associated with not being attended by a skilled birth attendant when giving birth (2 × 2 χ^2^, *p *= 0.0008) and was the third strongest predictor of giving birth without a skilled birth attendant after the number of children the woman had and level of formal education she had received [15].

Hence, illiteracy is a well-established barrier to receiving the recommended antenatal, giving birth and postnatal care, yet there is little documented about the lived experiences of illiterate pregnant women and mothers with newborns. Indeed, only one paper [25] providing qualitative data on this topic was found and none were found for rural regions or for LMICs. This is concerning since effective public health policy should be informed by local context [35–37]. What is it, for example, that renders illiteracy a risk factor for not receiving the recommended standard care during pregnancy, when giving birth, and after giving birth? In addition to missing data on lived experiences of illiterate women, specific obstacles to meeting recommended standards of perinatal care are sorely lacking for rural regions of LMICs. This study, consequently, was designed to begin to address this knowledge gap by using qualitative methods to capture the lived experiences related to care-seeking by illiterate, pregnant women and mothers in a rural region of an LMIC. Specifically, this study asks the following question: why don’t illiterate, pregnant woman in rural Tanzania access the recommended standard of maternal healthcare services?

## Methods

During design, data collection, and analysis, this study adhered to the consolidated criteria for reporting qualitative research (COREQ) when possible as outlined in Additional file 1: Appendix [38].

### Study area

**Fig. 1:**
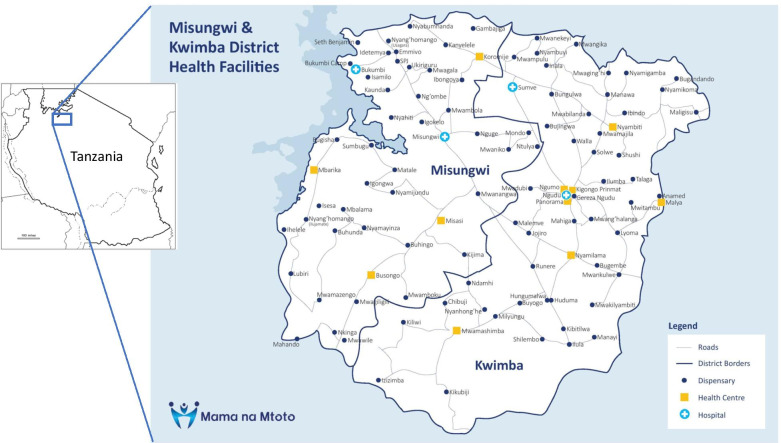
Misungwi District map. This map is our own creation

This research was conducted in Misungwi District located in Mwanza Region of Tanzania’s Lake Zone (Fig. 1). Misungwi District is rural, located 45 km from Mwanza city and at last census (2012) had a population of 351,607 [39]. Administratively, the district is sub-divided into 4 divisions, 20 wards, and 78 villages. In 2019, 91% of households in Misungwi District were ethnically Sukuma [15]. The Sukuma are a patrilineal society in which women are expected to take care of their husbands and children [40]. Those individuals included in this study were low-income, living in villages scattered throughout flatland terrain, and subsisting via the cultivation of maize, millet, rice, sweet potatoes and vegetables, cattle grazing on communal lands, and fishing. Most households surveyed in 2019 reported using firewood (83%) or charcoal (14%) for cooking fuel [15]. Sixty-eight percent of households owned livestock and 62% owned agricultural land [15]. Thirteen percent of households were connected to electricity, 57% owned a bicycle, and about 10% owned a mechanized form of transport [15]. Piped water, and advanced sanitation facilities are not common. Each of the four villages considered in this study had a primary school and attendance in primary school in Tanzania is compulsory. It is in school that students are taught to speak, read, and understand Swahili. While Swahili is the official language of the Tanzanian government and healthcare providers [41], it is the second language of most in Tanzania and is mainly learned at school, especially in rural communities. Despite the fact that primary school is compulsory in Tanzania, the country has a lack of a quality, formal education, especially in rural regions where the long distances to schools and insufficiently qualified and motivated teachers, a lack of teaching materials, textbooks, basic technology, and required financial contributions [42] are disincentives for some students [43, 44]. Additionally, Tanzanian girls are more likely to drop out of school than are boys due to their caretaking responsibilities [45]. In 2019, Misungwi District had 48 formal health facilities providing services for women giving birth. The district, along with others in the Lake Zone, has amongst the worst maternal, newborn, and child health indicators in the country [13], and is prioritized by government for maternal newborn health programming.

### Study sample

The study used Criterion-i [46], purposive sampling [47] to identify two rural divisions in Misungwi District. Purposive sampling is used in qualitative research for the identification and selection of information-rich cases [47]. Criterion-i, or criteria of inclusion sampling, is a purposive sampling strategy in which the sample is selected based on the assumption that they have knowledge and experience with the phenomenon of interest (i.e., accessing maternal healthcare) and will consequently be able to provide information that is both detailed and generalizable [46]. Villages were thus selected for inclusion by first ranking the four Misungwi District divisions surveyed in 2016 [15] on the basis of accessing antenatal, birth and postnatal care services, then selecting the two divisions, Mbarika and Inolelwa, with the lowest overall rates of accessing care. Four wards within Mbarika and Inolelwa were randomly selected for inclusion in this study [48] and within each ward, one village was randomly selected, for an overall total of four villages.

The illiterate women of reproductive age were recruited by first explaining the purpose and methods of the study to the village leaders and the village-based, community health workers (CHWs), then asking them to identify households most likely to have illiterate women of reproductive age. The village leaders and CHWs knew all residents of households in their catchments and had a sense of their literacy status. A second meeting was then held with all members of the village who wished to attend the focus group discussions (FGDs) to explain the purpose and methods of the study.

Subsequently, the households of potential research participants were visited by field researchers (DM, PN, VY, EN, & ZM) who explained the project. For those pregnant women or mothers of reproductive age who agreed to continue after learning more about the project, literacy was assessed by a standardized protocol [49]. Only women who could not read at all were eligible to participate in this study. This process was continued to attain a minimum sample size of 20 women in each village.

A total of 81 illiterate women who were either pregnant or had children and seven influential people, described below, were selected for inclusion in this study. Of these women, 7 (9%) were pregnant and had no children under-five years of age, 27 (33%) had one child under-five, 36 (44%) women had two children under-five, 9 (11%) had three children under-five, and no data on the number of children were available for 2 (3%) of the women. None of these women had attended school beyond the primary level. Thirteen in-depth interviews (IDIs) were conducted with the illiterate women and seven key-informant interviews (KIIs) were conducted with members of these communities who have first-hand experience with the decisions made by illiterate mothers concerning maternal and infant care. Of the 13 women who participated in the IDIs, all had attended at least one ANC and 11 had given birth with a skilled birth attendant. Interviews were conducted in the informants’ native language, Sukuma. All participants signed informed consent forms. No incentive was provided to the participants, other than refreshments, unless the participant incurred transportation costs to attend the interview(s), in which case transportation costs were refunded.

Those perceived to have some knowledge of women’s decisions concerning antenatal, birth and postnatal care services were also invited to participate in order to triangulate experiences of the women [50, 51]. These included opportunistically recruited CHWs and other healthcare providers in each of the four villages. CHWs are community members selected by their communities, trained using a national curriculum, and expected to voluntarily provide health promotion education and support emergency referral care to households in their community, especially to pregnant women and those who recently gave birth (e.g., if a CHW identifies an at-risk mother needing health care, the CHW would ‘refer’ the mother to a health facility). Furthermore, CHWs are neighbors, peers, and confidants of many of the women in their communities. Healthcare providers selected for interviews included nurses and clinical officers providing antenatal, birth, and postnatal services at health facilities. They were included as they have first-hand experience with decision making by women in the communities they serve. Potential CHWs or healthcare providers were excluded from participation in this study if they had not been active in their roles in the community for at least the prior six months. A total of two influential individuals were sought in each village. The field researchers met this goal in three of the four villages but were able to recruit only one influential individual in one of the villages.

### Data collection

This study is exploratory and utilizes a phenomenological approach to describe the lived experiences of illiterate women with regard to maternal healthcare [52–54]. Data were collected July–September, inclusive, 2018, in FGDs, IDIs and KIIs. In IDIs and KIIs, interviewers engaged in a probing conversation with the interviewee [55, 56] and used a single, semi-structured facilitator guide to maintain consistency across FGDs, IDIs, and KIIs (Additional files 2, 3 and 4: Appendices 2, 3 and 4) [57]. The only non-participants present in the FGDs or IDIs were infants of some of the participants. No non-participants were present during the KIIs. To ensure guiding questions resonated with participants, the facilitator guide was piloted twice in two similar villages in Tanzania’s Misungwi district. Questions and probes were refined after the pilots to better reflect the context of the region [58].

The morning after the women were selected for the study, FGDs were held with these women to gain an understanding of factors influencing their maternal healthcare-seeking decisions [59, 60]. FGDs were held in a community space chosen by the women. In FGDs, field researchers took a peripheral role to facilitate a group discussion between participants. Later that day, IDIs were held with individual, illiterate women selected at random from those who participated in the FGDs, to explore topics mentioned in the FGD in more depth [61]. As well, KIIs were held with the CHWs and healthcare providers. IDIs and KIIs were held in a location selected by the informant.

FGDs, KIIs, and IDIs were audio-recorded. Field researchers, comprised of a moderator, note-taker, and an observer, all fluent in Sukuma, facilitated the interviews. FGDs generally lasted 1–2 h; IDIs and KIIs lasted 45–60 min. Overall, 8 FGDs, two in each of the four villages, composed of 10–11 women each, for a total of 81 women, were conducted with follow-up IDIs completed with 13 (16%) of these women. Seven KIIs were conducted: 3 with CHWs and 4 with healthcare providers.

### Data analysis

Recorded Sukuma interviews were transcribed and translated directly and verbatim into Swahili as Swahili is the primary language of the Tanzanian team members. Transcriptions and translations were checked for accuracy by four of the Tanzanian researchers, fluent in Sukuma and Swahili, who did not conduct the original interviews or transcription/translation. Two additional Sukuma speakers conducted Sukuma source transcripts quality checks. Resulting Swahili transcripts were then translated to English by Tanzanian researchers fluent in English and Swahili. Interview data from FGDs, IDIs in the four communities with illiterate women, and KIIs with CHWs, and healthcare providers in the four communities were combined in the analysis and interpretation for two reasons: 1) each of these cohorts was asked the same questions and 2) this facilitated triangulation of the data from all four cohorts [51].

To provide a systematic account of the observed phenomena and transform interviews into a set of cohesive and meaningful categories, data were coded in four steps using NVivo (v. 12) [62] and, in step five, the credibility and validity of the findings were assessed. In step one, four randomly selected transcripts, including one IDI, one KII and two FGDs, were used to develop a coding template. Here, each of these transcripts was coded individually and the final codes subsequently agreed upon by DM, PN, and VY. In step two, four additional transcripts were selected at random and new codes were added if they did not fit with the initial codes. Step two resulted in the final codebook for the study. In step three, 18 additional transcripts were coded for a total of 26 (8 FGDs, 11KIIs and 7 IDIs) of the 28 transcripts coded, after which it was determined that saturation was reached; that is, new themes or sub-themes were unlikely to emerge from analysis of additional transcripts [63]. In step four, thematic analysis was used to collapse the codes into basic themes and subthemes [64]. In step five, data from FGDs and IDIs with the women, and IDIs with CHWs and healthcare providers in all four communities were triangulated to increase the credibility and validity of the findings [51].

## Results

The 81 illiterate women of reproductive age had a mean age of 32 ± 11 years, a mean of 1.7 ± 0.82 children under the age of 5 (Table 1). None of these respondents had any education. All were subsistence farmers engaged in farming small plots of land and 84% were married. All of these women spoke Sukuma, and none spoke or understood Swahili.

**Table 1:** Illiterate women of reproductive age sample demographics

Characteristics	n	%
Mothers' age^+^		
< 20	11	13.6
20–34	31	38.2
≥ 35	39	48.2
Marital status		
Married	68	84.0
Single	3	3.7
In-relationship	5	6.2
Widow	1	1.2
Divorced	4	4.9

Two content-driven themes related to antenatal care, birth and postnatal care access by women emerged from the analysis: 1) a communication gap arising from a) these women’s inability to read public health messaging documents provided by health facilities, and b) healthcare providers speaking Swahili, but not the local dialect, Sukuma, and 2) a dependency of these women on family and neighbors to negotiate their illiteracy and inability to speak Swahili. Each of these themes was apparent in transcripts from all participant categories. Moreover, there was no indication in the interviews that these women lacked health literacy; they knew that they should be attending the clinic for care but found it difficult to do so due to the communication gap.

### Communication gap: illiteracy

Participants in this study agreed that illiterate individuals exhibited poor adherence to health practices due to failure to understand health information, including appointment dates.

One illiterate woman of reproductive age explained that, “when we arrive at the health facility, we usually see a poster with medical advice. However, we usually do not understand what it says” (FGD). Another stated, “I usually do not know dates written on the card. I just take my baby to the health care facility for an injection. Then, the health care workers ask me to wait at the clinic. If there are no further vaccines, they tell me to go home. I [then] go back the next month” (IDI).

The CHWs were sensitized to the barriers faced by illiterate women, with one stating that “[illiterate] women are told to go back to the clinic within seven days but fail to return. This is probably due to her not knowing to read the information she was given on her card” (KII). Another pointed out that “You have to sit up and investigate. Often, when mothers come to the hospital and are registered, you can not recognize them all and we do not ask whether or not they can read and write. [As such,] it's hard to know who is illiterate” (KII).

### Communication gap: language

The inability of healthcare providers to speak Sukuma compromised their ability to help these women, who speak only Sukuma. Examples of the difficulties arising from this barrier were provided in the many statements by these women. An oft repeated point made by the women is illustrated in this statement: “We are unable to understand what the healthcare provider or CHW is telling us when she speaks Swahili as we do not know Swahili” (FGD). Due to this language barrier, most of the illiterate women expressed anxiety about visiting the clinic. Another common theme is manifested in the words of this illiterate woman of reproductive age: “We are afraid of going to the clinic because we are asked questions by the healthcare providers in Swahili, but we do not know Swahili” (FGD).

Healthcare providers and CHWs also identified this barrier. One health provider, for example stated that “illiterate women can’t speak Swahili, so are afraid of going to the hospitals since they are not sure if they will find a healthcare worker who can speak Sukuma” (KII). Another healthcare provider noted that “illiterate women are a real challenge in health education, one day I taught them about the improved Community Health Fund that was free for pregnant women and children under one year of age, but there was one woman who did not know how to read and write and did not understand Swahili. When she left the clinic, she went to tell others that…there is no free treatment for children or pregnant women” (KII). Consistent with these comments, a CHW stated that “the language barrier is a challenge for patients. Women fear going to the large, higher level, hospitals. There, they are not sure if they will find a healthcare provider who speaks Sukuma, leaving them unable to explain their condition. So, these are things that may lead to challenges for them when giving birth. But when they come to us, we speak Sukuma and just help them. …The patients really like speaking their native language. Even if she can read Swahili, some will not speak Swahili” (KII). Another CHW pointed out that “literate mothers ask a lot of questions so I can assess their understanding, but illiterate mothers just listen and don’t ask many questions, keeping their lack of understanding hidden from me so that they can just finish the appointment and leave” (KII).

### Negotiating illiteracy

The illiterate women in this study sought to negotiate their illiteracy via the assistance of literate neighbors or family members who could help them understand the public health messaging concerning appropriate antenatal, birth and postnatal care and infant development or remind them of appointment dates. Without such assistance they sometimes would not attend their recommended and scheduled appointments. As well, some illiterate women tried to keep track of dates via codes they developed. These strategies are apparent in the following statements by an illiterate woman: “We do not know anything until we get home and the information on our healthcare card is read for us. For example, if I have a child who knows how to read, he will help me to read the card” (FGD). Another illiterate woman added that “if there is a neighbor who is going to the clinic on the same date, she reminds me [and] we go together.” (FGD). Another illiterate woman noted that “when the date to go to the clinic arrives, family members tell me to go, so I go” (IDI) and “I get the assistance of a neighbor or a student at home to read and understand my healthcare card” (IDI). Many of the illiterate women stated the healthcare providers and CHWs endeavored to assist them. For example, an illiterate woman stated that “if you tell the healthcare providers the truth that you do not know how to write, they will know how to help you or they will tell you to find anyone who can help to read the information. If you do not know somebody to help you read the information, the healthcare provider will help you” (FGD).

The coping strategies of illiterate women were evident to the healthcare providers and CHWs. For example, one healthcare provider stated that “because illiterate women can’t read their healthcare card, they just rely on their neighbor’s date and only go to the clinic when their neighbors go” (KII). Along the same lines, a CHW pointed out that healthcare providers and CHWs encouraged illiterate women to rely upon literate family and neighbors to ensure they returned to the clinic on the proper date, stating, for example, that “what we're doing is just telling them the dates of the return and giving them a healthcare card with the date. If she forgets, we tell her to ask someone who knows how to read to remind her of the date when she should return to the clinic. This may be a child in school or a husband” (KII). Other healthcare providers and CHWs described other practices by illiterate women to ensure that they returned to clinic on the proper date. For instance, a healthcare provider stated that “some of the illiterate women fold pieces of fabric on the weekends to keep track of time. There was one woman here who was folding her fabric every Sunday and when the fourth Sunday had passed, she came to the clinic" (KII).

## Discussion

Globally, pregnant, illiterate women and illiterate mothers with newborns are less likely to receive the recommended standard care, with detrimental health sequelae. In Misungwi District, in comparison to literate women, illiterate women, who represent 35% of the women in this district, were significantly less likely to have at least four ANC visits or give birth under the care of a skilled birth attendant [15]. Why these women were less likely to access maternal care was unclear, requiring a deeper consideration of the link between these women’s perceptions, behaviors, and socio-cultural environment. Toward that end, this study endeavored to document the lived experience of illiterate women in rural Tanzania as it relates to the recommended standard of maternal healthcare.

Across the FGDs, KIIs, and IDIs with the illiterate women, CHWs, and healthcare providers in this study a consistent theme emerged: the inability of illiterate women to read, speak, or understand the language of healthcare providers was a formidable obstacle to illiterate women seeking maternal healthcare. Illiteracy is associated with two obstacles to receiving the recommended standard care and one coping strategy. First, illiterate women cannot understand written information provided to them by healthcare providers and CHWs. Clearly, this renders these women’s ability to recall appointment dates and read public health messaging a challenge. There is no apparent initiative on the part of healthcare providers or CHWs to determine which women are illiterate and illiterate women may be shy about admitting their illiteracy [23]. In the words of one CHW, determining who is illiterate is rendered more difficult by the fact that “illiterate mothers just listen and don’t ask many questions, keeping their lack of understanding hidden from me so that they can just finish the appointment and leave” (KII). If the CHW and healthcare provider are not aware of who is illiterate, their ability to address this issue is compromised. If they were aware of the woman’s ability to read and write, the CHW and healthcare providers did seek to assist the woman. For example, an illiterate woman noted that if you tell the CHW and healthcare provider that you are illiterate, they “will know how to help you or they will tell you to find anyone who can help to read the information” (FGD). Second, exacerbating their inability to read is the fact that the women in this study received no formal education, so did not learn to speak Swahili. These women therefore cannot understand those healthcare providers who do not speak their language, Sukuma. This inability to speak the language of those providing them with healthcare compromises their healthcare experiences. As noted above, a key benefit of ANC is the opportunity for healthcare providers to communicate with and support women during a critical period [7, 11, 12]. For example, exchanges between healthcare providers and pregnant women facilitate the relay pertinent information that may not be apparent to the healthcare providers, such as previous pregnancy complications, number of miscarriages or neonatal deaths, or a history of gestational diabetes. This benefit is lost for the women in this study. Even if they do receive the minimum standard of care when pregnant and with a newborn, the language barrier suggests that these illiterate women may gain little beyond the vaccines their newborns receive. Hence, the problem is larger than whether or not the mother can read or write; rather, illiteracy is a marker of ability to speak the language of healthcare providers. Furthermore, this problem may not be limited to illiterate women, as one CHW pointed out that “even if [a woman] can read Swahili, some will not speak Swahili” (KII).

The CHWs speak both Sukuma and Swahili and could translate for the women. Indeed, CHWs indicated that they speak Sukuma with women from their community as much as possible, with one noting that “if you speak to them in their language, they can express themselves very well” (KII). However, translating the healthcare providers’ Swahili for the illiterate women may not be feasible as CHWs are generally not present during ante- or post-natal care visits or when a woman gives birth with a skilled birth attendant.

These findings are consistent with the robust body of work noted above that finds a strong association between women's formal education and child health elsewhere [13, 26–33]. The current study considers the association between the level of formal education and use of healthcare services more deeply. Here, the driver for not receiving the recommended maternal healthcare is neither the lack of formal education nor the lack of understanding about the need for such care, generally, but, specifically, is the inability to understand the language of both healthcare providers and inability to read public health documents due to the lack of formal education.

The illiterate women in this study did not report feeling stigma about their inability to read or write, contrary to other research [23–25]. Within social groups, stigma is believed to function to reinforce power inequities, maintain social norms and avoidance of disease [65]. As regards norms, stigma is argued to be tightly tied to a society's ideas of how things should be [66] and arises when a phenomenon is perceived as a threat to material goods or symbolic goods such as beliefs, values, or ideology [67]. Thirty-five percent of the women surveyed in Misungwi District are illiterate [15]. As illiteracy is not uncommon in these communities and may have little bearing on a woman’s productivity as a subsistence farmer, it may not be perceived as a threat to norms and material and symbolic goods so may not be stigmatized. That said, the fact that the CHWs and healthcare providers stated that illiterate women did not reveal their illiteracy may indicate that these women do feel stigmatized and, as such, were remiss to admit feeling stigmatized in the FGDs or KIIs. If they do feel stigmatized for their illiteracy, this would very likely be a barrier to healthcare seeking [66].

The agency of these illiterate women is manifest in their efforts to negotiate their illiteracy via assistance from their relatives or neighbors to help them to understand public health messaging and to remind them of appointment dates written in their healthcare card. While these actions may well serve their purpose for some, being illiterate was the most important risk factor for failure to attend at least four ANC visits [15]. As above, even if an illiterate woman’s coping strategy gets her to her healthcare appointment on the assigned day, if the healthcare provider speaks only Swahili and there is nobody to translate Swahili into Sukuma, her healthcare experience may be compromised.

Interestingly, Tanzania’s 2015–2016 Demographic Health Survey (DHS) [13], finds that 67.4% of women aged 15–49 in the Lake Zone reported that they have serious problems accessing health care when they need it. In this DHS, women were asked if the problem accessing health care was due to insufficient money for treatment, distance to health facility, not wanting to go alone, and/or failure to obtain permission to go, and replied in the affirmative 52%, 47%, 30%, and 16% of the time, respectively [13]. A communication barrier is not noted in the DHS, likely reflecting the fact that these women were not asked if illiteracy or the ability to speak Swahili was a barrier and suggests that those responsible for the DHS may not have anticipated communication barriers.

### Strengths and limitations

Focus groups and interviews carried out with the protection of anonymity uncovered some of the perceptions of the women, CHWs and healthcare providers. These perceptions, arising from the informants’ socio-cultural situation are critical to healthcare-seeking decisions [35–37]. A further advantage of FGDs, IDIs, and KIIs is that they are resource effective, facilitating access to spontaneous views and a rich volume of data in a relatively short period of time. Moreover, these data were collected in the language of the informants by researchers with cultural sensitivity, then carefully translated by tri-lingual (Sukuma, Swahili, and English) researchers. A limitation of the study is the lack of data on the number of times each illiterate woman in this study accessed ante- or post-natal care or utilized a skilled birth attendant when giving birth. Hence, this study provides an exploration of barriers to maternal healthcare for women in these four communities overall, but not for specific individuals. Finally, it is important to keep in mind that this study likely masks a great deal of complexity. This cohort is not a static, isolated group. Rather, they exist and have agency within multidimensional socio-political networks that may not be readily apparent in a cross-sectional study.

## Conclusion

Public health messaging about the benefits of receiving the recommended care may have no impact on behavior and be misguided if local perspectives are not considered. The local perspective that emerges here is that the illiterate women in Misungwi district can neither understand the verbal communication of their healthcare providers nor can they read the information provided by them. Notably, these women do have health literacy and do appreciate the need to visit the community clinic. They know what they should do but gain little in attempts to access healthcare services.

Given that there are 128 languages in use in Tanzania, it is unrealistic to expect healthcare providers to learn the languages of each community they serve. Hence, in the short term, the needs of this at-risk population may be met by providing 1) a protocol for CHWs and healthcare providers to identify those who are illiterate or cannot speak Swahili; 2) a local individual with training in confidentiality or a cell-phone application to translate for those unable to speak Swahili; and 3) graphic public health messaging that does not require literacy. In the long term, this barrier may be overcome by ensuring that all Tanzanians have access to an education in which they would learn to read, write, and speak Swahili. A failure to address the obstacles to maternal care for these illiterate women will likely ensure that the maternal mortality ratio and neonatal mortality rate remain high in this region.

## Supplementary Information

**Additional file1: Appendix 1**. Table 2. Consolidated criteria for reporting qualitative studies (COREQ): 32-item checklist^1^.

**Additional file 2: Appendix 2** FGD Guide for illiterate women.

**Additional file 3: Appendix 3** IDI Guide.

**Additional file 4: Appendix 4** KII Guide.

## Data Availability

The data used in this study are available from the corresponding author on reasonable request.

## Abbreviations

ANC: Ante-natal care
CHW: Community-health worker
FGD: Focus group discussion
IDI: In-depth interview
KII: Key-informant interview
LMIC: Low-and-middle-income country
