# Supplementary material for: Why don’t illiterate women in rural, Northern Tanzania, access maternal healthcare?
Source: BMC Pregnancy Childbirth. 2021 Jun 28;21:452. doi: 10.1186/s12884-021-03906-2 (PMC8240192; doi:10.1186/s12884-021-03906-2)
Supplement: Supplementary file 1 — Additional file1: Appendix 1. Table 2. Consolidated criteria for reporting qualitative studies (COREQ): 32-item checklist1. [file 12884_2021_3906_MOESM1_ESM.docx]

**Appendix 1**

**Table 2: Consolidated criteria for reporting qualitative studies (COREQ): 32-item checklist**^[[1]](#footnote-1)^

| **Item** | **Guide questions/description** | **Reply** |
| --- | --- | --- |
| **Domain 1: Research team and reflexivity** | |  |
| *Personal Characteristics* |  |  |
| 1. Inter viewer/facilitator | Which author/s conducted the interviews and focus groups? | DM, PN, VY, EN, & ZM |
| 2. Credentials | What were the researcher’s credentials? (e.g., PhD, MD) | DM, MD  PN, MscDS  VY, MPH  RL, MD, PhD  RB, MD  EN, MD  ZM, MD, Misungwi District Medical Officer  MM, MPH  JLB, MD  WMW, PhD in anthropology |
| 3. Occupation | What was their occupation at the time of the study? | DM is a lecturer in the Department of Obstetrics & Gynecology, Catholic University of Health and Allied Sciences, Tanzania and an Obstetrician & Gynecologist Bugando Medical Centre, Tanzania.  PN is a lecturer in the School of Public Health, Catholic University of Health and Allied Sciences, Tanzania.  RL is a lecturer in the School of Nursing, Catholic University of Health and Allied Sciences, Tanzania.  RB is a paediatrician in the Department of Pediatrics, Catholic University of Health and Allied Sciences, Tanzania.  EN is a lecturer in the Catholic University of Allied and Health Sciences, Tanzania and obstetrician in the Bugando Medical Centre, Tanzania.  ZM is the District Medical Officer, Misungwi District, Tanzania.  MM is the Health System Strengthening Coordinator at Agriteam Canada Consulting Ltd.  JLB is a pediatrician and clinical associate professor. Departments of Pediatrics and Community Health Sciences at the Cumming School of Medicine, University of Calgary, Canada.  WW is an associate professor in the Department of Anthropology & Archaeology and adjunct associate professor in the Department of Community Health Sciences, University of Calgary, Canada. |
| 4. Gender | Was the researcher male or female? | DM, male. PN, female. VY, female. RL, female. RB, male. ZM, male. MM, female. JLB, female. WMW, male. |
| 5. Experience and training | What experience or training did the researcher have? | Please see “Credentials” and “Occupation” above. |
| *Relationship with participants* |  |  |
| 6. Relationship established | Was a relationship established prior to study commencement? | A relationship was established prior to data collection. |
| 7. Participant knowledge of the interviewer | What did the participants know about the researcher? e.g. personal goals, reasons for doing the research | The participants knew that the field researchers (DM, PN, VY, EN, & ZM) were from the Catholic University of Health and Allied Sciences and were interested in understanding those factors that influenced their decision to seek maternal healthcare. |
| 8. Interviewer characteristics | What characteristics were reported about the inter viewer/facilitator? e.g. Bias, assumptions, reasons and interests in the research topic | The participants knew that the field researchers (DM, PN, VY, EN, & ZM) were from the Catholic University of Health and Allied Sciences and were interested in understanding those factors that influenced their decision to seek maternal healthcare. |
| **Domain 2: study design** |  |  |
| *Theoretical framework* |  |  |
| 9. Methodological orientation and Theory | What methodological orientation was stated to underpin the study? e.g. grounded theory, discourse analysis, ethnography, phenomenology, content analysis | This study is exploratory and utilizes a phenomenological approach to describe the lived experiences of illiterate women with regard to maternal healthcare. |
| *Participant selection* |  |  |
| 10. Sampling | How were participants selected? | Purposive and convenience sampling was used to select the participants. |
| 11. Method of approach | How were participants approached? | Participants were approached face-to-face. |
| 12. Sample size | How many participants were in the study? | In this study, there were 81 illiterate women who were either pregnant or had children, three Community Health Workers, and four healthcare providers. |
| 13. Non-participation | How many people refused to participate or dropped out? Reasons? | Research subjects joined if they wished to join. None dropped out after joining. |
| *Setting* |  |  |
| 14. Setting of data collection | Where was the data collected? | Focus group discussions were held in a community space chosen by the participants. In-depth and key-informant interviews were held in a location selected by the informant. |
| 15. Presence of non-participants | Was anyone else present besides the participants and researchers? | The only non-participants present were a few of the infants of the participating women. |
| 16. Description of sample | What are the important characteristics of the sample? | The important characteristics of the sample are provided in the manuscript in both the methods and Table 1. |
| *Data collection* |  |  |
| 17. Interview guide | Were questions, prompts, guides provided by the authors? Was it pilot tested? | Questions prompts and guides were provided by the interviewers and the interview was pilot tested. The interview guides are provided in Appendices 2-4. |
| 18. Repeat interviews | Were repeat inter views carried out? If yes, how many? | Repeat interviews were not carried out. |
| 19. Audio/visual recording | Did the research use audio or visual recording to collect the data? | The research used audio recording to collect the data |
| 20. Field notes | Were field notes made during and/or after the interviews or focus groups? | Field notes were made during the interviews and focus groups. |
| 21. Duration | What is the duration of the interviews or focus group? | Durations:  Focus-group discussions: 1-2 hours.  In-depth interviews & Key-informant interviews: 45-60 minutes. |
| 22. Data saturation | Is data saturation discussed? | Data saturation is discussed. |
| 23. Transcripts returned | Were transcripts returned to participants for comment and/or correction? | We did not return transcripts to participants for comment or correction because they were anonymized at the time of transcription. |
| **Domain 3: analysis and findings** |  |  |
| *Data analysis* |  |  |
| 24. Number of data coders | How many data coders coded the data? | Three data coders (DM, PN, & VY) coded the data. |
| 25. Description of the coding tree | Do authors provide a description of the coding tree? | A description of the coding tree is not provided in the article. |
| 26. Derivation of themes | Were themes identified in advance or derived from the data? | Themes were derived from the data. |
| 27. Software | What software, if applicable, was used to manage the data? | NVivo (v. 12) was used to manage the data. |
| 28. Participant checking | Did participants provide feedback on the findings? | We did not perform member checking. |
| *Reporting* |  |  |
| 29. Quotations presented | Are participant quotations presented to illustrate the themes/findings? Is each quotation identified? e.g. participant number | Participant quotations are presented to illustrate the themes/findings and each quotation is identified. |
| 30. Data and findings consistent | Is there consistency between the data presented and the findings? | There is consistency between the data presented and the findings. |
| 31. Clarity of major themes | Are major themes clearly presented in the findings? | Major themes are clearly presented in the findings. |
| 32. Clarity of minor themes | Is there a description of diverse cases or discussion of minor themes? | There is a description of diverse cases or discussion of minor themes |

1. Checklist adapted from: Tong A, Sainsbury P, Craig J. Consolidated criteria for reporting qualitative research (COREQ): a 32-item checklist for interviews and focus groups. *International Journal for Quality in Health Care*. 2007. Volume 19, Number 6: pp. 349 – 357 [↑](#footnote-ref-1)
