# Supplementary material for: Why don’t illiterate women in rural, Northern Tanzania, access maternal healthcare?
Source: BMC Pregnancy Childbirth. 2021 Jun 28;21:452. doi: 10.1186/s12884-021-03906-2 (PMC8240192; doi:10.1186/s12884-021-03906-2)
Supplement: Supplementary file 3 — Additional file 3: Appendix 3 IDI Guide. [file 12884_2021_3906_MOESM3_ESM.docx]

**Appendix 3**

**IDI Guide**

**FGD IDNO** |___|___|___|___| **Date** (DD/MM/YY) |___|___/___|___/___|___|

**Facilitator Initials** |___|___|___| **Note-taker Initials** |___|___|___|

**Participant sub-group:** (circle): / community leaders /Influential people

**Audio file**: |___|___|___|

**Community number:**|___|___|

| **INTRODUCTION**  **I am ______________________________ (facilitator) from the Catholic University of Health and Allied Sciences Bugando-Mwanza working for Mama na Mtoto Project.**  **I am ______________________________ (note-taker) from the Catholic University of Health and Allied Sciences Bugando-Mwanza working for Mama na Mtoto Project.**  • Ask the participant to introduce herself    **We are with you today to learn from you about how and why mothers utilize or do not utilize ante-natal care, delivery and post-natal care services. We are especially curious to know what makes it easier or harder for women in this community to utilize ante-natal care, delivery and post-natal care services and whether or not there are people in your community who are good at persuading you and other mothers to utilize these services. We will do this in in-depth Interview with you to recall things that influenced your utilization of ante-natal care, delivery and post-natal care services. Your ideas as participant are very important for us to gain your ideas, opinions, and experiences. We will use this information to inform stakeholders and policy makers to improve health services and policies related to maternal new-born and child health in your community.**  **Before beginning our conversation, we ask you to go through the consent form with us. If, after going through the form, you agree to participate, we will ask you to sign two copies of the consent form. We will give one copy to you to keep for your reference and will keep one copy for our records.**  **Review consent form with participant. Be sure to cover all the following points:**   - General purpose of the study - Study procedures - Benefits of participation - Risks of participation - Confidentiality - Voluntary participation - Questions   **Obtain two signed consent forms before proceeding. One copy is for the participant and one copy is for the research team.**  **Demographic data will be captured using another form**  **Define the rules that will guide us during this discussion, including the following:**   - Ask the participant to define ground rules, for example: - It is important for us to hear all sides of an issue – the positive and the negative. - Confidentiality is assured. “What is shared in the room stays in the room.” - Any questions? - Check position and functioning of tape recorder - Check participant’s consent to participate and be recorded - Refreshments will be served after the discussion   **Now I am going to introduce topics about experiences on women during pregnancy, delivery, and after the birth of your child. In particular, we will discuss the utilization of ante-natal care, delivery, and post-natal care services. In each case, I will ask you whether or not women access the particular type of health care services, what factors made it difficult and easier for them to access this type of healthcare, whether anybody in your community persuaded women to utilize the healthcare, and what you think might make it easier for them to access the type of healthcare.** | |
| --- | --- |
| **Domain** | **Topic, Questions, and *Probes*** |
| Warm up questions | - How are doing with farming? - At your free time, what do you do for you to relax? |
| Identify those cohorts who are less likely to access ANC, delivery, and PNC services. | **1.0 Let us begin by sharing the utilization of ante-natal care services.**  **1.0a** In your opinion, what groups of women do you think are less likely to access ANC services?  **1.0b** Are there any other groups you think are less likely to access ANC services?  *Facilitator should probe other groups not mentioned by the participants. (widows, single women, far from health facility, women with more children).*  **1.1 Now we are discussing the use of delivery services at a Health Facility.**  **1.1a** In your opinion, what groups of women do you think are less likely to access delivery services at health facilities?  **1.1b** What other groups do you think are less likely to access delivery services at health facilities?  *Facilitator should probe other groups not mentioned by the participants. (widows, single women, far from health facility, women with more children).*  **1.2 Now we are discussing the utilization of post-natal care services.**  **1.2a** In your opinion, what groups of women do you think are less likely to access PNC services?  **1.2b** What other groups do you think are less likely to access PNC services?  *Facilitator should probe other groups not mentioned by the participants. (widows, single women, far from health facility, women with more children).* |
| Barriers, Enablers, & Influencers | **Since you have an experience with ante-natal care, delivery, and post-natal care services, you have a lot to share with us about the factors which affect our your use of these services.**  **2.1 Let us begin by sharing your experiences with ANC.**  **2.1a** What made it **harder** for you to access ANC services?  **2.1b** Can you tell us more about how and why those things you mentioned made it harder for you to access ANC?  *Facilitator should probe other things not mentioned by the participant (e.g. distance to health facility, village by laws, cost, supplies, clothes, etc.)*  **2.1d** What made it **easier** for you to access ANC services?  2.1e Can you share an example?  **2.1f** Can you tell us more about how and why those things you mentioned made it easier for you to access ANC?  *Facilitator should probe other things not mentioned by the participant (e.g. distance to health facility, village by laws, cost, supplies, clothes, etc.)*  **2.1h** Did any members of your community increase your willingness to attend ANC?  **2.1i** Can you tell us about how did these people increased your willingness or that of others to attend ANC?  **2.1j** Is there anything these people could do to more effectively persuade you attend ANC?  **2.2 Now let us share your experiences concerning delivery.**  **2.2a** Where do you deliver in this community deliver?  *Probe, if not mentioned, Health Facility, Home, TBA*  **2.2b** Are there any challenges that prevent you from accessing Health Facility delivery?  **2.2c** Can you share an example?  **2.2d** How would you suggest that we make delivery at a Health Facility easier for you?  **2.2e** What other experiences have you experienced with delivery?  **2.2f** Can you share who encourages you to opt for home or TBA delivery rather than delivery in a Health Facility?  **2.2g**  Can anyone share more about other motivations?  *Probe about infrastructure, waiting time, perception, supplies*, *clothes.*  **2.2h** What else do you think about delivery?  *Probe about home delivery, TBA delivery if not already mentioned and discussed by the participants.*  **2.2i** Did any members of your community increase your willingness in your community to have delivery at a Health Facility?  **2.2j** Can you tell us about how did these people increased your willingness or that of others to have your delivery at a Health Facility?  **2.2k** Is there anything these people could do to more effectively persuade you to have your delivery at a Health Facility?  **2.3 Now let us share about your experiences with PNC.**  **2.3a** What made it **harder** for you to access PNC services?  **2.3b** Can you tell us more about how and why those things you mentioned made it harder for you to access PNC?  *Facilitator should probe other things not mentioned by the participants (e.g. distance to health facility, village by laws, cost, supplies, clothes, etc.)*  **2.3c** Can you share an example?  **2.3d** What made it **easier** for you to access PNC services?  **2.3e** Can you share an example?  **2.3f** Can you tell us more about how and why those things you mentioned made it easier for you to access PNC?  *Facilitator should probe other things not mentioned by the participant (e.g. distance to health facility, village by laws, cost, supplies, clothes, etc.)*  **2.3h** Did any members of your community increase your willingness and that of other women in your community to attend PNC?  *Probes: Is there anybody else who increased the willingness of women in your community to attend PNC such as family, relatives, health workers, community health workers, or leaders?*  **2.3i** Can you tell us about how did these people increased your willingness or that of others to attend PNC?  **2.3j** Is there anything these people could do to more effectively persuade you to have you to attend PNC? |
| **Recommendation** | As we mentioned earlier, the goal of this project is to increase the use of ANC, delivery, and PNC services in your community. An important way to do this is to provide recommendations to policy makers and others involved with these services. Because you are the a mother in the community that the healthcare providers seek to serve, your thoughts here are critical to help make these services more attainable for people in your community. Earlier you discussed those things that make it harder or easier for you to utilize ANC, delivery, and PNC services. Now we’d like to revisit these points to generate recommendations for the community and healthcare providers to increase the use of these services.  **2.4a** ANC: You mentioned earlier that _______ makes it **harder** for you to access ANC and _______ makes it **easier or more likely** for you to access ANC. With these points in mind, what do you recommend that your community, including the health facility and influencers, do to make it easier or more likely that you will attend ANC?  **2.4b** Delivery: You mentioned earlier that _______ makes it **harder** for you to deliver at a Health Facility and _______ makes it **easier or more likely** for you to deliver at a Health Facility. With these points in mind, what do you recommend that your community, including the health facility and influencers, do to make it easier or more likely that you will deliver at a Health Facility?  **2.4c** PNC: You mentioned earlier that _______ makes it **harder** for you to access PNC and _______ makes it **easier or more likely** for you to access PNC. With these points in mind, what do you recommend that your community, including the health facility and influencers, do to make it easier or more likely that you will attend PNC? |
| **Wrap up** | **2.5a** Do you have any questions or recommendation regarding this discussion?   - *Thank the participant for her time in attendance and participation.* |
